# Wellness Coaching: Practices and Preferences Among Life Plan Community Residents and Staff

**DOI:** 10.1093/geroni/igaa057.1213

**Published:** 2020-12-16

**Authors:** Matthew Fullen, Jennifer Smith, Janis Sayer, Philip Clarke, Connie Tomlin

**Affiliations:** 1 Virginia Tech, Blacksburg, Virginia, United States; 2 Mather Institute, Evanston, Illinois, United States; 3 Wake Forest University, Terre Haute, Indiana, United States

## Abstract

Wellness coaching, a process in which a coach and client partner together to address the client’s wellness goals, can increase motivation and develop skills to enhance wellness and lifestyle balance among older adults. To understand wellness preferences among Life Plan Community residents, we surveyed a total of 447 residents from 10 Life Plan Communities. Participants were asked about perceptions of wellness, wellness activity preferences, motivators and barriers to participation in wellness activities, and wellness coaching program preferences. Twenty employees in wellness-related or leadership roles also completed a survey. To enhance our understanding of these perspectives on wellness coaching, nine residents and four employees participated in follow-up interviews. Among the study findings, the majority of resident respondents expressed an interest in improving almost all domains of wellness. Forty percent (40%) of participants said they were extremely or moderately likely to try wellness coaching, and about one-half (51%) said they believed they would benefit from wellness coaching. Staff indicated interest in implementing a wellness coaching program in their community, with 74% reporting at least a moderate likelihood of implementing a program if led by a staff member. Results indicated that emotional and vocational wellness programs were offered and attended less frequently than other types of wellness programs, which suggests that wellness coaching could help to address the need for more programming in these areas. In addition, findings suggested that implementation requires resident input to ensure buy-in. The survey results informed the development of recommendations for a resident wellness coaching program.

